# Cultural Adaptation and Validation of an Arabic Version of the Modified Harris Hip Score

**DOI:** 10.7759/cureus.14478

**Published:** 2021-04-13

**Authors:** Abdullah N Al-Qahtani, Osama A Alsumari, Hussam S Al Angari, Yazeed N Alqahtani, Rakan A Almogbel, Abdullah A AlTurki

**Affiliations:** 1 Orthopaedics, College of Medicine, King Abdulaziz Medical City, Riyadh, SAU; 2 Orthopaedics, King Abdulaziz Medical City, Riyadh, SAU; 3 Medicine, College of Medicine, King Saud bin Abdulaziz University for Health Sciences, Riyadh, SAU; 4 Orthopaedics, King Abdulaziz Medical City Riyadh, Riyadh, SAU; 5 Medicine, College of Science and Health Profession, King Saud bin Abdulaziz University for Health Sciences, Riyadh, SAU; 6 Orthopaedic Surgery, King Abdulaziz Medical City, Riyadh, SAU

**Keywords:** arabic, mhhs, modified harris hip score, questionnaire, translation

## Abstract

Context

Although the Modified Harris Hip Score (MHHS) is used worldwide, it has not been translated into Arabic or validated for use among Arabic populations.

Aim

This study aimed to translate the MHHS into Arabic and to culturally adapt and validate the Arabic version.

Design

A cross-sectional study of the MHHS was performed.

Methods

The MHHS was translated into Arabic using forward-backward translation. A total of 183 adults who could speak and read Arabic completed the questionnaire. Cronbach’s alpha was used to assess internal consistency with respect to the total and subscale scores. Pearson’s correlation coefficient was used to evaluate associations between the total scores, and the subscales and intersubscales. Test-retest reliability was assessed using the intraclass correlation coefficient (ICC). The Kaiser-Meyer-Olkin value was determined before principal component analysis to evaluate the validity of the construct and the reliability of the data, and correlations among the score items were estimated.

Results

All the participants understood the questions. The ICCs for the total score, function subscale, and pain subscale were 0.936, 0.936 and 0.893, respectively. Cronbach’s alpha was acceptable for the total score (0.792) and good for the function subscale (0.895). The total score and the function (r = 0.976; p < 0.001) and pain (r = 0.971; p < 0.001) subscales correlated significantly. Regarding score validity, all the MHHS items correlated with the total score (p < 0.001).

Conclusions

The reliability and validity of the Arabic version of the MHHS was demonstrated. The MHHS can be used to assess hip pathology among adults in Saudi Arabia.

## Introduction

Osteoarthritis frequently affects the hip, with the disease only affecting the knee with more prevalence. Due to its high incidence, many functional outcome measures are used to assess the level of hip joint disability. These tools evaluate the effectiveness of different treatment modalities such as the Harris Hip Score (HHS) and its modified version (MHHS). The MHHS is simple to administer, widely used, and excellent for detecting changes in pain and function pre- and post-operatively [1]. Hence, this study aimed to translate the MHHS into Arabic and to validate and culturally adapt the tool for use in the Arab population.

## Materials and methods

Study design

A cross-sectional study was conducted over a four-month period from November 2019 to March 2020. We collected data from three major medical centers. We administered the MHHS (HHS without the deformity and range of movements subdomains) to individuals who met the following inclusion criterion: adults of either sex with hip pain or hip disabilities who could read and understand Arabic. Our exclusion criteria were as follows: patients with unstable medical conditions and unable to participate and non-Arabic-speaking patients. Additionally, we provided the selected individuals with informed consents.

Patients

The sample size was determined from a literature review, and the internal consistency of the MHHS was measured using Cronbach’s alpha, which varies from 0.93 to 0.98 [2]. Assuming very similar performance within our study population, we required a minimum sample size of 67 participants to estimate the internal consistency of the MHHS with 95% confidence and 5% precision [3]. A total of 183 patients were enrolled in this study.

Modified Harris Hip Score

We used the protocol established by the World Health Organization [4] to translate and adapt the MHHS. We started with forward translation and used two independent translators for this step, which generated two versions of the translated MHHS. The two versions were combined into one by an experienced orthopedic consultant. Then, two independent translators on an expert panel conducted a backward translation, and we pretested the instrument on a group of 20 patients. The feedback from the pretest group led to some changes in the text that ensured greater acceptability among patients. The amended version was the final version of the tool. We started collecting data using the translated MHHS immediately after the tool was validated by our research center. As in the original HHS, the MHHS has a 100-point scale, and the total scores are classified as poor (<70), reasonable (70-80), good (80-90), or excellent (90-100) [5]. After subtraction of the deformity and range of motion domains, the total score became 91; hence, to get a maximum score of 100 in our research, we multiplied the score of each patient by a factor of 1.1.

Statistical analyses

SPSS software Version 25.0 (IBM Corp., Armonk, NY, USA) was used to analyze the data. The categorical data are presented as frequencies and percentages, and the continuous data are presented as means and standard deviations.

Cronbach’s alpha was used to assess the internal consistency of the MHHS with respect to the total score and the subscales. The correlations between the total score, and the subscales and intersubscales were evaluated using Pearson’s correlation analysis. Test-retest reliability was assessed using the intraclass correlation coefficient (ICC). The Kaiser-Meyer-Olkin value, which should be < 0.60, was determined as a measure of sampling adequacy before principal component analysis of the construct validity of the Arabic MHHS. Relationships between the items in the Arabic MMHS were tested using Pearson’s correlation coefficients, of which many should be >0.3. Values of p < 0.05 were considered statistically significant.

## Results

This study included 183 participants. The feedback of the participants indicated that there were no difficulties in understanding the items in the MHHS; 42 participants tested the MHHS for reliability, and the ICCs for the total score, function subscale, and pain subscale were 0.936, 0.936, and 0.893, respectively. Table 1 presents the data describing the reliability of the MHHS.

**Table 1:** Test-retest reliability of the Modified Harris Hip Score ICC, intraclass correlation coefficient; SD, standard deviation

	Test (n = 183)		Retest (n = 42)		ICC (95% confidence interval)	Alpha coefficient
	Mean	SD	Mean	SD		
Total score	53.45	26.64	57.64	23.47	0.936 (0.885–0.965)	0.967
Function subscale	25.50	13.45	27.30	12.76	0.936 (0.884–0.965)	0.967
Pain subscale	30.33	12.75	27.95	14.39	0.893 (0.809–0.941)	0.893

Cronbach’s alpha values were 0.792 and 0.895 for the total score and function subscale, respectively. Table 2 presents the correlation coefficients for the MHHS (p < 0.001). Statistically significant correlations were evident between the total score and the function (r = 0.976; p < 0.001) and pain (r = 0.971; p < 0.001) subscales. The correlation between the function and pain subscales was also statistically significant (r = 0.897; p < 0.001).

**Table 2:** Correlation between the items within the Modified Harris Hip Score

	Pain	Distance walked	Activities—shoes, socks	Public transport	Support	Limp	Stairs	Sitting
Pain	1.000	.857	0.651	0.684	0.782	0.870	0.743	0.773
Distance walked	0.857	1.000	0.709	0.725	0.768	0.875	0.758	0.751
Activities—shoes, socks	0.651	0.709	1.000	0.584	0.725	0.703	0.691	0.634
Public transport	0.684	0.725	0.584	1.000	0.619	0.732	0.569	0.643
Support	0.782	0.768	0.725	0.619	1.000	0.730	0.766	0.640
Limp	0.870	0.875	0.703	0.732	0.730	1.000	0.733	0.789
Stairs	0.743	0.758	0.691	0.569	0.766	0.733	1.000	0.663
Sitting	0.773	0.751	0.634	0.643	0.640	0.789	0.663	1.000

Regarding the validity of the score, all of the MHHS items correlated significantly with the total score (p < 0.001). Table 3 presents the correlation coefficients. The suitability of the data was assessed before analysis of the principal components; the correlation coefficients for many score items were >0.3, and the Kaiser-Meyer-Olkin value was 0.937 (0.6). Furthermore, these values exceeded the recommended values for analysis of the principal components, which showed that the pain component of the MHHS had an eigenvalue of >1 (6.06), which exceeded the Kaiser criterion. Additionally, a screen plot also revealed a clear break in the curve after the pain component, and thus it was decided to retain the pain component. However, as only one component was retained, the procedure could not provide a solution.

**Table 3:** Correlations between the Modified Harris Hip Score items and the total score R, correlation coefficient

Item	Total score, r
Pain	0.971
Distance walked	0.921
Activities—shoes, socks	0.760
Public transport	0.740
Support	0.864
Limp	0.924
Stairs	0.820
Sitting	0.824

## Discussion

The HHS is one of the most commonly used scoring systems to assess the condition of patients’ hips [6]. However, a high inter-observer bias led to criticism of the physical examination component of the HHS, but this has little impact on the overall score [7,8]. Removing the physical examination component from the HHS led to the development of the MHHS, and this version has been used extensively because its administration requires less medical expertise, its completion is simpler, and its scores can be calculated remotely because patients do not have to physically attend clinics [9].

In this study, we investigated the reliability and validity of the Arabic version of the MHHS. The study participants did not experience difficulties in comprehending and answering the questions in the MHHS. The results of this study showed that this version of MHHS was internally consistent, with a significant association between the scale and its subscales and excellent temporal reliability; furthermore, component analysis showed that the pain component had the largest weight in this scale.

Cronbach’s alpha coefficient was 0.792, which indicates a moderate level of internal consistency according to the recommended range of 0.70-0.95, and the score’s items were homogeneous, interdependent, and nonredundant [10]. Similar results have been reported from studies using versions of the MHHS developed in Turkey, Greece, and India [11-13]. However, Hung et al. reported poor consistency of MHHS with a Cronbach alpha coefficient of 0.41 [14].

Regarding test-retest reliability, 42 participants were re-evaluated after the initial assessment and the ICC was 0.936, which indicates excellent temporal reliability of the Arabic version of the MHHS, and this version is deemed adequate for use in clinical practice [15]. Similarly, evaluations of the MHHS translated for use in Turkey, Greece, and India generated ICCs > 0.90 [11-13]. Reports from two other studies have shown similar findings [16,17]. In contrast, Hinman et al. reported a low ICC value of 0.76 [18]. Additionally, we conducted factor analysis for the Arabic version of the score, which revealed that the pain component had the largest weightage in the total score of the MHHS; however, this component could not be retained statistically because it was the only component with large weightage.

In summary, we developed an Arabic version of the MHHS and evaluated its reliability and validity using a variety of methods. Psychometric evidence suggests that it is sufficiently reliable and valid for use in clinical settings. One of the limitations of this study was that it was a cross-sectional study, and the factor of change of responsiveness overtime was not assessed. Another limitation was that it was conducted in three centers; therefore, its results might not be representative of the entire Arabic-speaking population. However, the strength of this study comes from its relatively large sample size. Future studies are recommended to assess the change in responsiveness over time, assess the reliability and consistency of this scale in a larger and more diverse Arabic-speaking population, and confirm the results from this study.

## Conclusions

A psychometric analysis of our study’s data demonstrated that the Arabic version of the MHHS is a valid and reliable tool that can be used to assess different hip pathologies in adult populations in Saudi Arabia. The translated MHHS can now be used in clinical practice and for research purposes.
